# Persistent transgene expression in peripheral tissues one year post intravenous and intramuscular administration of AAV vectors containing the alphaherpesvirus latency-associated promoter 2

**DOI:** 10.3389/fviro.2024.1379991

**Published:** 2024-03-28

**Authors:** Carola J. Maturana, Esteban A. Engel

**Affiliations:** Princeton Neuroscience Institute, Princeton University, Princeton, NJ, United States

**Keywords:** gene therapy, peripheral tissue, promoter, transduction, AAV8, AAV9, gene delivery, latency-associated promoter

## Abstract

Significant progress has been made in enhancing recombinant adeno-associated virus (rAAV) for clinical investigation. Despite its versatility as a gene delivery platform, the inherent packaging constraint of 4.7 kb imposes restrictions on the range of diseases it can address. In this context, we present findings of an exceptionally compact and long-term promoter that facilitates the expression of larger genes compared to conventional promoters. This compact promoter originated from the genome of the alphaherpesvirus pseudorabies virus, latency-associated promoter 2 (LAP2, 404 bp). Promoter driving an mCherry reporter was packaged into single strand (ss) AAV8 and AAV9 vectors and injected into adult C57BL/6 mice at a dose of 5 × 1011 vg/mouse by single intravenous or intramuscular administration. An ssAAV8 and ssAAV9 vector with elongation factor-1α promoter (EF1α, 1264 bp) was injected side-by-side for comparison. After 400 days, we sacrificed the mice and examined mCherry expression in liver, kidney, heart, lung, spleen, pancreas, skeletal muscle, and brain. We found that LAP2 exhibited robust transgene expression across a wide range of cells and tissues comparable to the larger EF1α, which is currently recognized as a rather potent and ubiquitous promoter. The AAV8-LAP2 and AAV9-LAP2 constructs displayed strong transduction and transcription in liver, kidney, and skeletal muscle on both route of administration. However, no expression was detected in the heart, lung, spleen, pancreas, and brain. The outcomes of our investigation propose the viability of LAP2 for gene therapy applications demanding the expression of large or multiple therapeutic genes following a single viralvector administration.

## 1 Introduction

Gene delivery via recombinant adeno-associated virus (rAAV) stands as a cornerstone in both research and clinical applications due to its enduring transgene expression and safety profile. This aspect is essential for the efficacy and acceptance of AAV gene therapy as a reliable treatment option for various genetic and acquired diseases. Sustained expression ensures long-term therapeutic benefits, especially for chronic diseases that require persistent treatment. This reduces the need for frequent re-administration, improving patient compliance and reducing the healthcare burden. Besides, a good safety profile minimizes the risk of adverse effects, such as immune responses or off-target effects, which could compromise patient health or the success of the therapy. Ensuring safety is essential for gaining regulatory approval and public trust in gene therapy treatments. Despite these advantages, the challenge persists in precisely targeting specific tissues and cell types (1-3). While tissue-specific promoters exist, ubiquitous promoters offer robust and enduring transgene expression, potentially surpassing their specialized counterparts (4, 5).

Clinical trials often employ AAV vectors with strong but potentially repressible promoters such as chicken beta-actin, miniCBA (800 bp) or the hybrid of CBA promoter and MVM intron, CBh (800 bp), which could require high vector doses that can lead to toxicity and exacerbated immune responses (5-7). Despite potential drawbacks, such as the need for high vector doses leading to toxicity, the consistent and potent expression driven by these promoters is often deemed crucial for therapeutic efficacy, particularly in diseases requiring high levels of transgene expression for optimal outcomes. Additionally, their wellcharacterized regulatory elements and widespread use in preclinical research contribute to their selection in clinical trials, facilitating regulatory approval and streamlining development processes (8). To enhance transgene expression without exceeding the packaging capacity of AAV, researchers have introduced transcription factor binding sites and viral regulatory elements such as enhancers or introns (9-11). To overcome issues of transcriptional silencing of therapeutic genes and to achieve high expression levels without the need for high vector dose, we identified a short, potent, and persistent promoter in the genome of the alphaherpesvirus pseudorabies virus (PrV) (12). This constitutive PrV latency-associated promoter (LAP), comprising LAP1 and LAP2 tandem sequences, orchestrates sustained transcription in neurons, evading epigenetic silencing during latency (12, 13). Previous studies indicated LAP2 as a potential determinant of PrV infectivity in peripheral organs (14). Our earlier research showcased robust transgene expression in mouse peripheral tissues following systemic AAV-LAP2 administration, regardless of PrV infection, 30 days post-administration. Comparing LAP2 (404 bp) with the larger EF1α promoter (1264 bp) demonstrated LAP2 potency equivalent to EF1α, regardless of AAV serotype or route of administration (ROA), despite its smaller size by 66% (15). In this study, we conducted thorough comparative research on two promoters in adult mice over an extended time period. We packaged the mCherry fluorescent reporter gene driven by the LAP2 or EF1α promoter into single stranded (ss) AAV8 and AAV9 vectors and injected them into mouse at a dose of 5 × 10^11^ gc by single intravenous (IV) or intramuscular (IM) administration. After 400 days (i.e., > one year), we sacrificed the mice and examined mCherry expression in liver, kidney, heart, lung, spleen, pancreas, skeletal muscle, and brain. We observed that AAV8-LAP2 and AAV9-LAP2 sustain potent and lasting mCherry expression in the liver, kidney, and skeletal muscle, comparable to the larger EF1α promoter. Broad tropism and varied cellular expression patterns of AAV-mCherry genome were detected in these tissues. RNA *in situ* hybridization confirmed the efficient transcriptional activity of the promoter, suggesting the potential of LAP2-based gene therapy vectors to achieve long-term, ubiquitous transgene expression at high level. These findings support the development of new LAP2-based gene therapy vectors for use in clinical conditions to achieve potent, broad, and ubiquitous transgene expression.

## 2 Materials and methods

### 2.1 Production of AAV vectors

**FIGURE 1:**
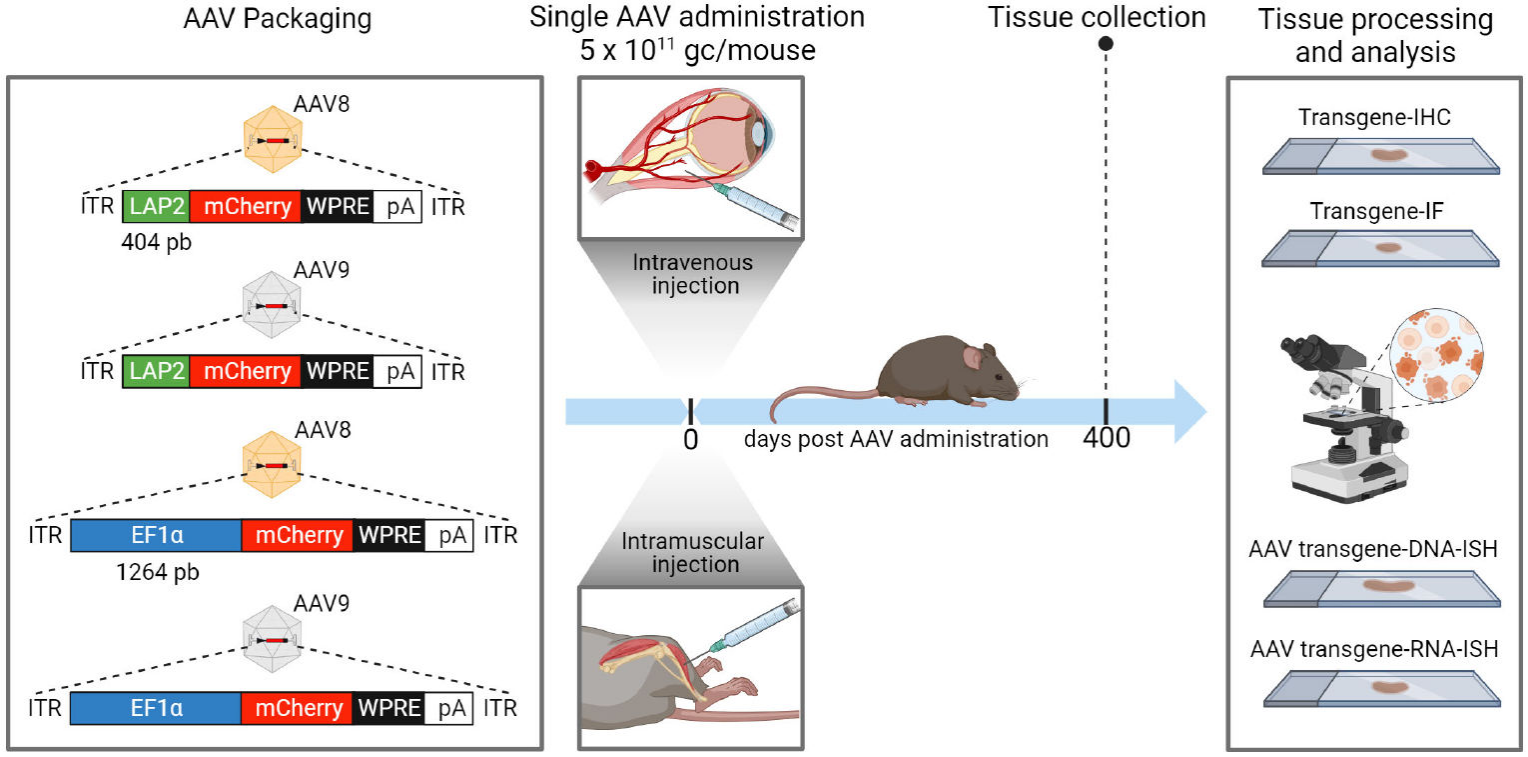
Adeno-associated virus (AAV) vector construction and workflow of *in vivo* dosing, tissue collection and imaging analysis. An illustration of the expression cassettes of four vectors designed to transcribe mCherry fluorescent reporter under the control of LAP2 (404 bp) or EF1± (1264 bp) promoters. All plasmids contain the woodchuck hepatitis virus post-transcriptional regulatory element (WPRE) and the human growth hormone polyadenylation (pA) signal. Each plasmid was packaged into AAV8 or AAV9 serotype. All AAV vectors were administered either intravenously (IV) into the retro-orbital sinus or intramuscularly (IM) into the tibialis anterior of male C57BL/6J wild-type mice at a dose of 5 × 1011 genome copies (gc) per mouse. Peripheral tissues, including the liver, kidney, and quadriceps muscles, were collected for analysis 400 days after AAV administration. Tissues were processed and sectioned for immunohistochemistry (IHC), immunofluorescence (IF), and DNA/RNA in *situ* hybridization (ISH) studies. ITR, inverted terminal repeat. Created with BioRender.com.

All AAV vector plasmids were generated as described previously (15). Briefly, the AAV-LAP2 plasmid (Addgene plasmid #159524), was constructed by double digestion of vector pAAV-EF1α-mCherry with MluI and BamHI followed by subcloning of the appropriate LAP fragment upstream of the mCherry reporter gene, flanked by AAV2 ITRs. pAAV-EF1α-mCherry was a gift from Karl Deisseroth (Addgene plasmid #114470). AAV plasmids included the gene encoding for the mCherry fluorescent protein driven by the LAP2 promoter or the EF1a promoter as a control. AAV plasmids were packaged into ssAAV8 or ssAAV9 serotypes that carried the woodchuck hepatitis virus post-transcriptional regulatory element (WPRE) and the human growth hormone polyadenylation signal sequence (Figure 1). Virus titers (genome copies [gc] per milliliter) were determined using a TaqMan^®^ real-time polymerase chain reaction assay (Thermo Fisher Scientific, Rockford, IL, USA) with primers/probe targeting WPRE (forward primer: 5’-GGCTGTTG GGCACTGACAA-3’; reverse primer: 5’-CCAAGGAAAGGA CGATGATTTC-3’; probe: 5’-TCCGTGGTGTTGTCG-3’) (16, 17). All AAV vectors were produced by the Princeton Neuroscience Institute (PNI) Viral Core Facility (Princeton University).

### 2.2 Animals and vector administration

Mice studies were performed according to the Institutional Animal Care and Use Committee of Princeton University (protocol 1947-19). Five-week-old male C57BL/6J mice were purchased from Jackson Laboratories (Bar Harbor, ME, USA). The mice were maintained on a 12-hr light/dark cycle with access to food and water ad libitum. For IV vector administration, 100 μl of the AAV preparation was injected unilaterally into the retro-orbital sinus. For IM administration, 50 μl of the AAV preparation was injected unilaterally into the tibialis anterior. Each mouse received 5 × 10^11^ gc of each vector. Mice were euthanized 400 days after inoculation via intraperitoneal injection of ketamine (400 mg/kg) and xylazine (50 mg/kg) followed by perfusion with 4% paraformaldehyde (Fisher Scientific, Waltham, MA, USA).

### 2.3 Histological processing

Liver, kidney, heart, lung, spleen, pancreas, and skeletal muscle samples were post-fixed for 24 hr at 4°C. The whole brain was postfixed for 2 hours at room temperature (RT) (18). All tissues were dehydrated in a sucrose gradient as previously described (19, 20). Histology was performed by HistoWiz, Inc. (histowiz.com) via standard protocols. Samples were processed, embedded in paraffin, and sectioned at 5 μm for *in situ* hybridization (ISH) or block-frozen and sectioned at 10 μm for immunohistochemistry (IHC) and immunofluorescence (IF) evaluation.

### 2.4 Immunohistochemistry and immunofluorescence staining

Tissue sections were analyzed by chromogenic IHC targeting mCherry and counterstained with hematoxylin. All staining was performed by HistoWiz Inc using the Leica Bond RX Automated Stainer (Leica Microsystems, Wetzlar, Germany). IF analysis was performed as previously described (19). Briefly, sections were blocked with 3% bovine serum albumin, 2% donkey serum, and 0.5% Triton X-100 (Sigma-Aldrich, St. Louis, MO, USA) for 1 hr at RT. The tissue sections were then incubated overnight at 4°C with primary antibodies, followed by incubation with the appropriate secondary antibodies for 1 hr at RT. Sections were mounted with an antifade solution containing 4’,6-diamidino-2-phenylindole (DAPI) (Vector Laboratories, Newark, CA, USA) to stain the nuclear DNA. Antibodies from Thermo Fisher Scientific were used: Alexa Fluor 488 phalloidin (1:200), Alexa Fluor 488 donkey anti-mouse IgG (1:1000), and Alexa Fluor 647 donkey anti-rat IgG (1:1000). Antilaminin gamma 1 (A5, Novus Biologicals, Centennial, CO, USA) was used at a 1:400 dilution.

### 2.5 RNA/DNA ISH

ISH detection of messenger RNA (mRNA) encoding mCherry was performed using an RNAscope^®^ 2.5 High-Definition Red assay (Advanced Cell Diagnostics [ACD], Newark, CA, USA). Briefly, tissue sections were boiled for 15 min in target-retrieval solution and pretreated with proteinase-plus solution for 30 min at 40°C. The pretreated tissues were incubated with RNAscope^®^ target probes (ACD, catalog no. 431201) for 2 hr at 40°C and counterstained with hematoxylin. To detect mCherry DNA, the protocol was adapted according to the manufacturer’s recommendations and included an incubation with RNase A (5 mg/ml; QIAGEN, Hilden, Germany) for 30 min at 40°C immediately before hybridization with the DNAscope^®^ target probes (ACD, catalog no. 1107941-C1) via overnight incubation at 40°C. The tissues were then counterstained with hematoxylin.

### 2.6 Image analysis

Whole-slide scanning at 40x magnification was conducted on liver, kidney, heart, lung, spleen, pancreas, and skeletal muscle tissues using an Aperio^®^ AT2 scanner (Leica Biosystems). Morphometry was performed using a HALO^®^ image analysis platform (Indica Labs, Albuquerque, NM, USA) by HistoWiz, Inc. Whole brain sections and fluorescent images were captured on a Leica SP8-LSCM confocal microscope (20x) (21). Image reconstructions of z-stacks and intensity projection images were generated using ImageJ (https://imagej.nih.gov/ij/) and quantified with QuPath 0.3.0 software (https://qupath.github.io). The fluorescence intensity was calculated by adapting the corrected total cell fluorescence formula (22, 23). The average number of dots per cell in both DNAscope^®^ and RNAscope^®^ assays was calculated using the ACD scoring criteria: 0: no staining or <1 dot/10 cells; 1: 1–3 dots/cell; 2: 4–9 dots/cell and/or few to no dot clusters; 3: 10–15 dots/cell and/or <10% of the dots in clusters; 4: >15 dots/cell and/or >10% of the dots in clusters.

### 2.7 Statistical analysis

Data are represented as mean ± standard error of the mean (SEM). For data sets with multiple groups, significance was determined by one-way analysis of variance (ANOVA) with Tukey’s post-test. A p-value <0.05 was considered statistically significant. Statistical analysis was performed using GraphPad Prism 10 software (GraphPad, La Jolla, CA, USA).

## 3 Results

To examine the potency and persistence of AAV-LAP2 vectormediated gene expression, we packaged the fluorescent reporter mCherry downstream of LAP2 into AAV8 and AAV9 capsids. We compared the transcription and translation efficacy of LAP2 with that of the ubiquitous EF1± promoter. The AAV vectors were injected either IV into the retro-orbital sinus or by local IM administration into the tibialis anterior at doses of 5 × 10^11^ gc per mouse. Tissues were collected for histological and morphometric analyses at 13 months (400 days) after AAV administration (Figure 1).

### 3.1 Comparative evaluation of AAV-LAP2 and AAV-EF1a promoters for long-term transgene expression: tissue-specific insights and route of administration influence

To evaluate the extent of AAV-LAP2-mediated long-term transgene expression, we examined mCherry expression in liver, kidney, heart, lung, pancreas, spleen, skeletal muscle, and brain mouse tissues using IHC. Administration of AAV-LAP2 resulted in potent mCherry expression in the liver, kidney, and skeletal muscle 400 days after either local or systemic administration at levels similar to those observed with the EF1α promoter (Supplementary Figure 1). However, heart, lung, spleen, and pancreas showed no transgene expression from either promoter (Supplementary Figure 2). In the brain, we observed remarkably faint mCherry expression in mice injected with LAP2 or EF1a promoters (Supplementary Figure 3).

**FIGURE 2:**
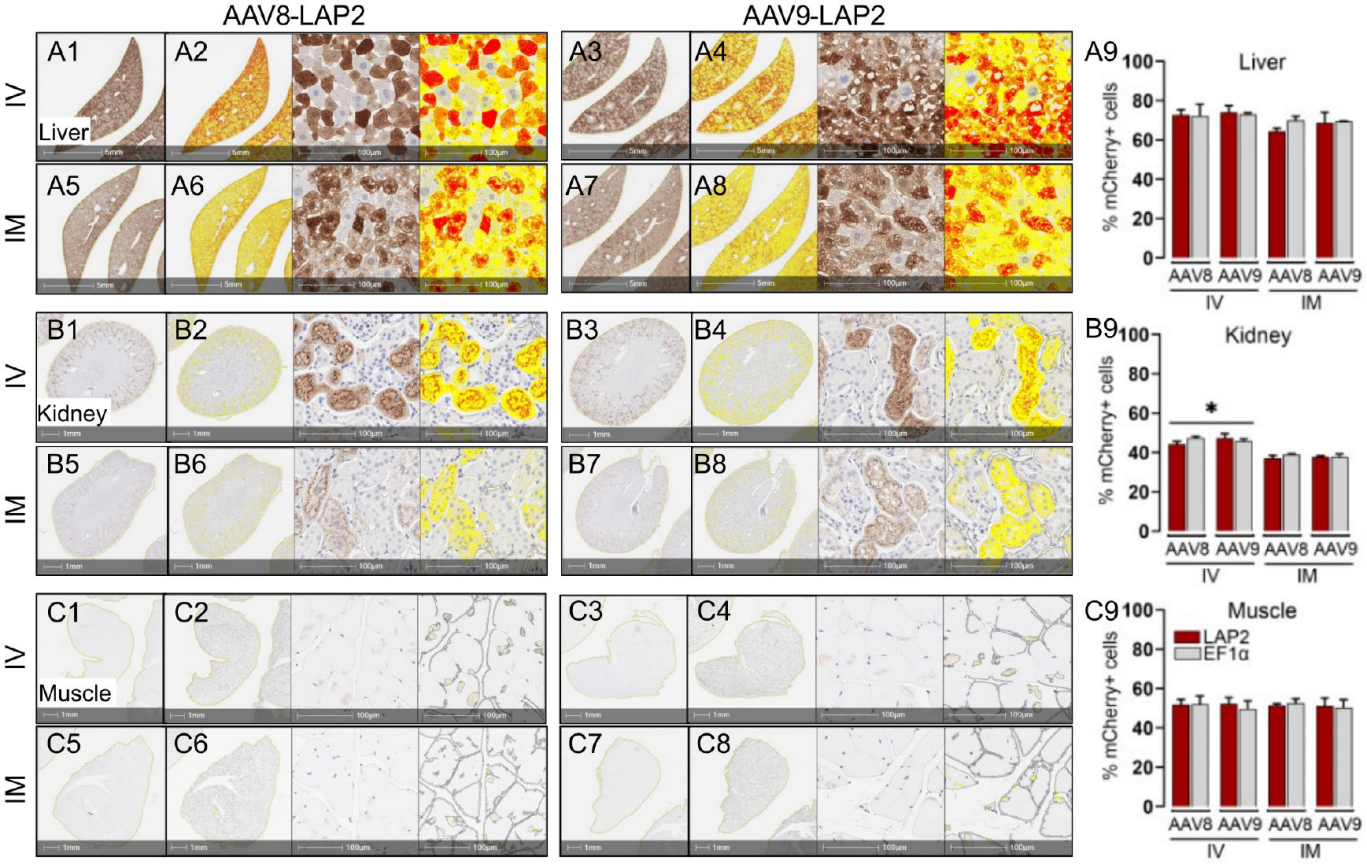
Transgene expression driven by the LAP2 is strong and stable for long term in tissues *in vivo* after one year. Representative images of immunohistochemically stained mCherry (brown) in **(A)** liver, **(B)** kidney, and **(C)** skeletal muscle tissue after intravenous (IV) **(A1–A4, B1–B4, C1–C4)** or intramuscular (IM) **(A5–A8, B5–B8, C5–C8)** administration of AAV8-LAP2 or AAV9-LAP2 vectors. Higher-magnification images corresponding to the morphometric analysis used to quantify mCherry staining are also shown. Intensity of low, medium and high mCherry expressions are represented by yellow, orange and red respectively. Scale bars, 5 mm, 1 mm, and 100 μm as shown. **(A9, B9, C9)** Quantification of mCherry-positive nuclei per unit area in mice tissues dosed with AAV-LAP2 (red) or AAV-EF1± (grey). Data are represented mean + SEM; *p <0.033 compared with the alternative ROA; n = 3 mice per condition, with three tissue sections analyzed per mouse.

**FIGURE 3:**
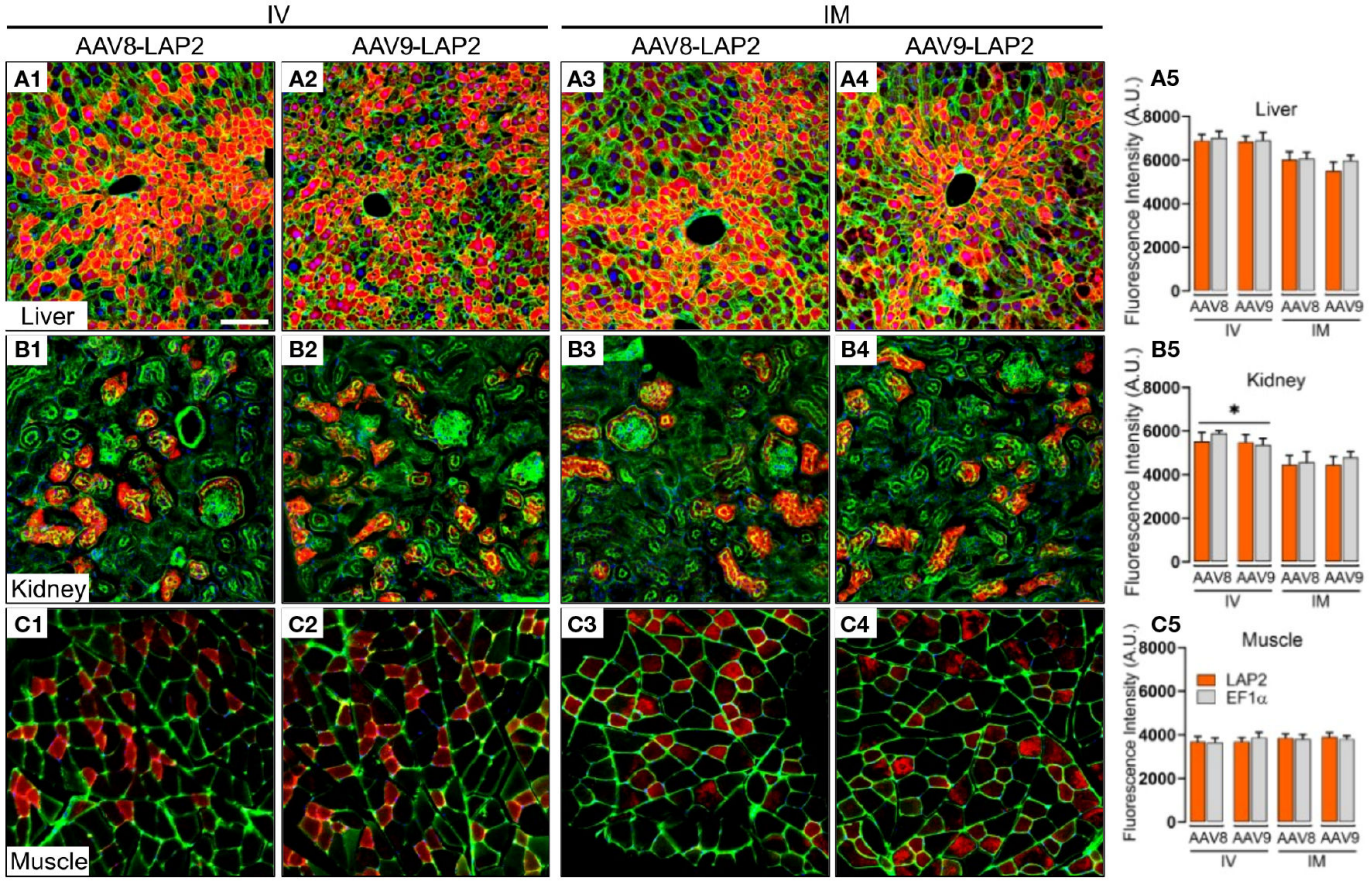
Widespread, long-term expression of the mCherry transgene after AAV8-LAP2 orAAV9-LAP2 dosing is independent of IV or IM routes of administration. Representative confocal images of fluorescent mCherry signal (red) in **(A)** liver, **(B)** kidney, and **(C)** skeletal muscle tissue 400 days after intravenous (IV) **(A1–A2, B1–B2, C1–C2)** or intramuscular (IM) **(A3–A4, B3–B4, C3–C4)** administration of AAV8-LAP2 or AAV9-LAP2. The liver and kidney sections were stained with phalloidin (green), and skeletal muscle was stained with anti-laminin (green). Nuclei were counterstained with DAPI (blue). All images are stacked confocal sections. Scale bar, 100 ¼m. **(A5, B5, C5)** Quantification of the fluorescence intensity of the native mCherry signal driven by AAV-LAP2 (orange) and AAV-EF1± (grey). Data are represented as mean ± SEM; *p < 0.033 compared with the alternative route of administration; n = 3 mice per condition. Three tissue sections were analyzed from each mouse. Significance was determined by one-way ANOVA with Tukey’s post-hoc test. A.U., arbitrary units.

The staining pattern in the liver was almost entirely restricted to the central vein, with no differences observed between the two promoters. Similarly, mCherry expression in kidney sections of animals dosed with either promoter was restricted to the cortex and enriched in the glomerulus, proximal tubule, and distal convoluted tubules. Cross-sections of stained skeletal muscle revealed a mosaic-type expression pattern, with similar potencies observed with both promoters (Figures 2A-C). The expression of mCherry was quantified as the percentage of total positive-staining cells per square millimeter of tissue. We observed over 40% of mCherry positive cells in the kidney, 50% in skeletal muscle, and 75% in the liver from mice inoculated with either AAV-LAP2 or AAV-EF1± (Figures 2A9, B9, C9). Interestingly, AAV-LAP2 and AAV-EF1± delivered by the systemic route generated significantly higher levels of mCherry compared with that observed in response to local administration (Figure 2B9). This was further confirmed by IF studies showing that AAV-LAP2-driven transgene expression was significatively enriched in the kidney after IV administration (Figures 3B1-5). Our results revealed no statistically significant differences between promoters, AAV serotypes, or ROA in the liver and muscle (Figures 3A1-5, C1-5). Significance was determined by one-way analysis of variance (ANOVA) with Tukey’s post-test. A pvalue <0.05 was considered statistically significant.

### 3.2 Long-term spatial distribution and transcriptional activity analysis of AAV-LAP2 in liver, kidney, and skeletal muscle reveals broad tropism and diverse cell-type expression patterns

**FIGURE 4:**
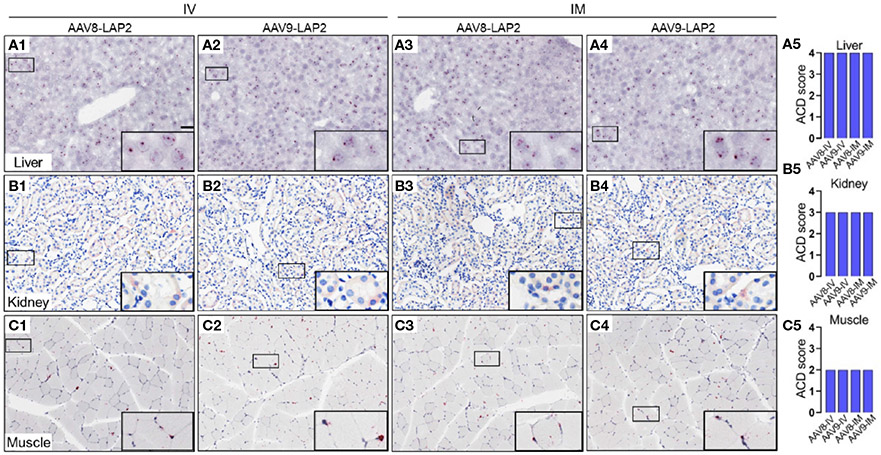
Spatial and cellular distribution of the AAV vector DNA in the liver, kidney, and skeletal muscle following IV and IM administration. In *situ* hybridization (ISH) was performed using the DNAscope^®^ Multiplex Assay. DNA encoding for the mCherry transgene was visualized by alkaline phosphatase-Fast Red detection. Nuclei were counterstained with hematoxylin. AAV DNA was identified by an mCherry sense probe to analyze **(A)** liver, **(B)** kidney, and **(C)** skeletal muscle tissue 400 days after intravenous (IV) **(A1-A2, B1-B2, C1-C2)** or intramuscular (IM) **(A3–A4, B3–B4, C3–C4)** administration of AAV8-LAP2 or AAV9-LAP2. Scale bar, 100 μm. **(A5, B5, C5)** Quantitative evaluation of average AAV DNA detected in the liver, kidney, and skeletal muscle using Advanced Cell Diagnostics (ACD) scoring criteria (see Methods for more details).

**FIGURE 5:**
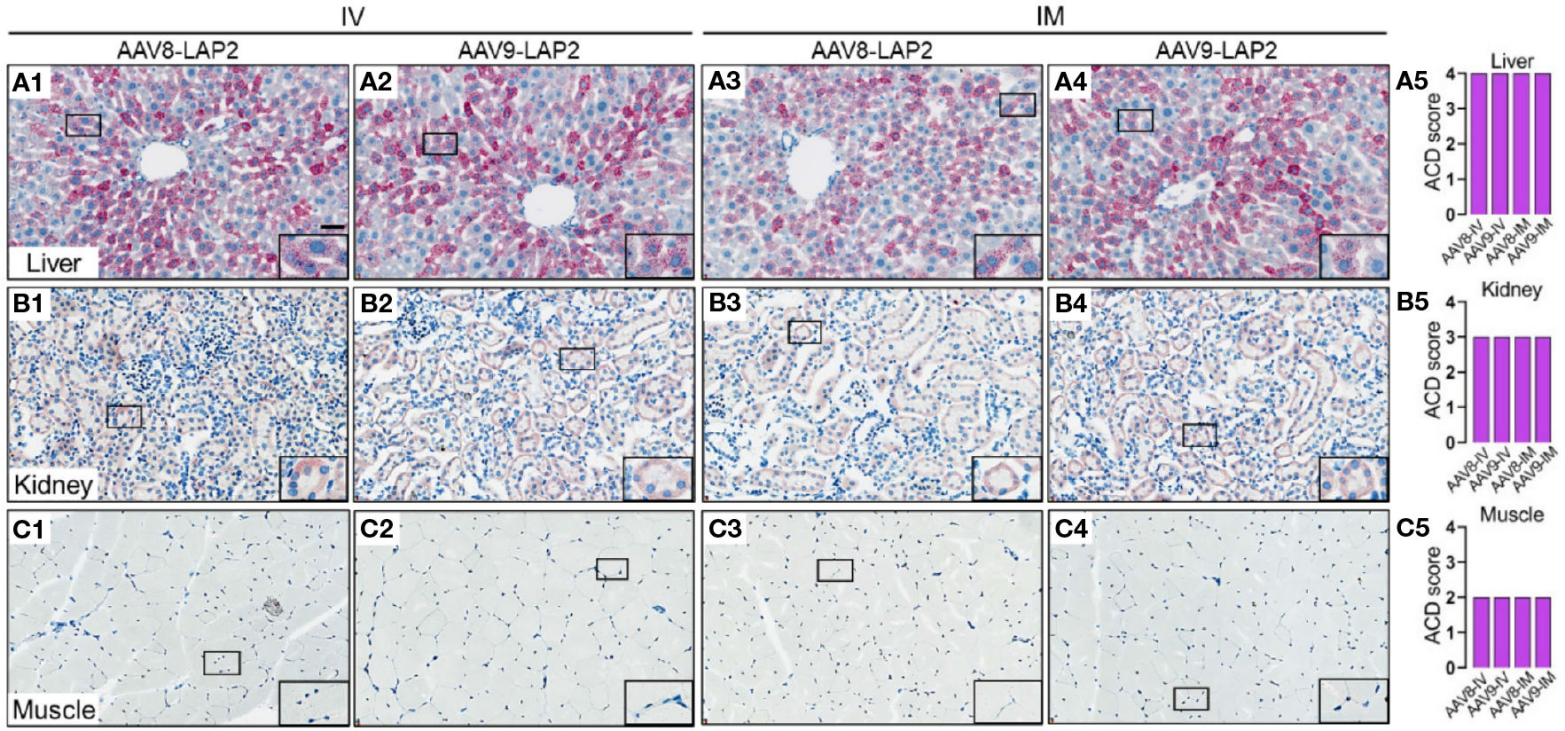
LAP2 shows strong transcriptional activity in the liver, kidney, and skeletal muscle after one year post IV or IM administration. Quantitative analysis was performed using the RNAscope^®^ Multiplex Assay. mCherry mRNA was visualized by alkaline phosphatase-Fast Red detection. Nuclei were counterstained with hematoxylin. AAV RNA was identified by an mCherry antisense probe to evaluate **(A)** liver, **(B)** kidney, and **(C)** skeletal muscle tissue 400 days after intravenous (IV) **(A1–A2, B1–B2, C1–C2)** or intramuscular (IM) **(A3–A4, B3–B4, C3–C4)** administration of AAV8-LAP2 or AAV9-LAP2. Scale bar, 100 ¼m. **(A5, B5, C5)** Quantitative evaluation of average AAV RNA detected in the liver, kidney, and skeletal muscle was performed with the Advanced Cell Diagnostics (ACD) scoring criteria.

To assess the spatial and cellular distribution of AAV-LAP2 and evaluate promoter-driven transcriptional activity in the liver, kidney, and skeletal muscle, we conducted quantitative analysis employing modified DNAscope^®^ and RNAscope^®^ techniques to detect AAV genomes and mCherry mRNA, respectively, 400 days post local or systemic vector administration. Quantitative assessments of AAV DNA and mRNA were performed using ACD scoring criteria (see Material and Methods). We identified mCherry mRNA that were concentrated in foci in all regions of the liver (Figure 4A) and found considerably higher levels of mCherry transcript in the liver than in the kidney or skeletal muscle (Figures 4B, C). The liver, kidney, and muscle were assigned ACD scores of 4,3, and 2, respectively on a scale from 0 to 4 (Figures 4A5, B5, C5). However, by combining our transgene detection with ACD scoring criteria, we determined that the liver exhibited >15 dots per cell with >10% of the dots found in clusters (Figures 5A1-5). The kidney showed an average of 10–15 dots per cell with <10% of the dots localized in clusters (Figures 5B1-5). By contrast, we detected an average of 4–9 dots per cell in skeletal muscle with few to no dot clusters (Figures 5C1-5).

## 4 Discussion

AAV vectors are a well-established and clinically validated platform for gene therapies (24). In most cases, the efficacy of these therapies requires potent and persistent transgene expression in one or more target tissues or cell-types. Gene transfer and expression can be optimized according to the specific disease paradigm and are largely determined by the AAV serotype, dose, route of administration, and type of regulatory elements (e.g., promoters) (25-27). Ubiquitous promoters are typically more potent than tissue-specific promoters (5). While several wellknown constitutive promoters, such as cytomegalovirus (CMV; 800 bp), CMV enhancer fused to the chicken beta-actin promoter (CAG, 1.7 kb), or EF1± promote efficient and long-lasting transgene expression, their large size restricts the payload capacity that can be devoted to the therapeutic transgene(s) (8). Thus, the development of small, potent, and persistent gene promoters is of paramount importance for successful gene therapy. In this study, we report the results of experiments to further validate the short, constitutive, and long-lasting LAP2 promoter derived from PrV. We demonstrate robust and persistent transgene expression facilitated by AAV8-LAP2 and AAV9-LAP2 across mouse liver, kidney, and skeletal muscle for more than a year post-IV or IM delivery. LAP2 exhibits comparable efficacy to the larger EF1± promoter. As a result, we observe extensive biodistribution and consistently elevated levels of transgene-derived mRNA and protein driven by AAV8-LAP2 and AAV9-LAP2 in these tissues. Notably, we previously showed robust transgene expression from AAV-LAP2 following IV administration 30 days post-injection, in contrast to local delivery. However, heart, lung, spleen, and pancreas showed no transgene expression from either promoter. We examined brain tissue from mice injected with AAV9-LAP2 and AAV9-EF1±, as the AAV9 serotype is known to penetrate the blood-brain barrier (BBB) with low efficiency. In previous studies, we demonstrated robust and specific LAP2 expression in all neurons in mice injected with the PHP.eB serotype after six months (19). Importantly, PHP.eB is an AAV9-based capsid that was engineered for efficient BBB crossing (28). As expected, we observed extremely weak mCherry expression in the brains of mice injected with AAV9-LAP2 or AAV9-EF1± promoters. The limited ability of AAV9 serotype compared to PHP.eB to cross the BBB led to poor brain transduction and weak transgene expression in the brain (28).

The liver assumes a pivotal role in metabolizing circulating substances, potentially resulting in elevated vector accumulation owing to inherent processing and clearance mechanisms, irrespective of the injection pathway (29). Our findings in liver are consistent with early studies demonstrating efficient transgene expression form AAV2/8-TBG (liver-specific thyroxin binding globulin (TBG) promoter, 800 bp) in cats and AAV8- and AAV9-TBG in dogs for up to one year (30, 31). These results suggest that a lack of immune response contributes to AAV vector persistence.

Several studies have been performed with different AAV serotypes and ROA in combination with cell-specific promoters to improve transgene expression in kidneys (32). However, low kidney transduction has been reported with systemic administration of AAV9 vectors (33, 34). We previously demonstrated that AAV8-LAP2 and AAV9-LAP2 drive potent transgene expression in kidneys at 30 days post IV dosing. Particularly, side-by-side comparisons showed that IV dosing of either AAV8-LAP2 or AAV9-LAP2 resulted in potent transgene expression in the renal cortex compared to local administration after one year. The kidneys receive significant blood supply, which passes through them to be filtered for urine excretion. This makes them more accessible to systemically administered AAV vectors, increasing the likelihood of successful transduction relative to IM ROA (35).

Consistently observed mosaic expression patterns in skeletal muscle across AAV-LAP2 serotypes and ROA can be attributed to robust mCherry expression in myofibers, as previously reported (15). Specially, enduring transgene expression was observed in type 2 muscle fibers following local administration at 30 days (15) maintaining comparability across both ROA after a year. Intramuscular injection might lead to immune responses within the muscle itself, potentially affecting transduction efficiency over time, whereas systemic administration could distribute vectors to numerous muscle fibers, mitigating immune responses and sustaining higher expression levels (36) Our findings are in line with pioneering research by Xiao et al. (37), who demonstrated successful long-term AAV-mediated gene transfer to skeletal muscles over 12 months through intravenous dosing of AAV2-CMV (cytomegalovirus promoter, 800 bp). Similarly, our study indicates enduring AAV-LAP2 transduction in skeletal muscle using a promoter as potent as CMV or EF1±, albeit significantly smaller, to allow for additional payload capacity for therapeutic transgenes.

In summary, our findings provide evidence that the LAP2 promoter can drive persistent, potent, and widespread transgene expression in the liver, kidney, and skeletal muscle, after either IV or IM administration. A direct comparison of the LAP2 and EF1± promoters revealed that, despite its smaller size, LAP2 is as potent as EF1± regardless of the AAV serotype or ROA. Reducing promoter size increases the payload capacity of the AAV vector. This optimization could potentially allow for the inclusion of supplementary therapeutic genes, regulatory elements, or other functional components within the limited cargo capacity of AAV vectors. By maximizing the efficiency of gene delivery and expression, such advancements could enhance the therapeutic efficacy and versatility of AAV-based treatments, offering new opportunities for addressing complex diseases and improving patient outcomes.

This study builds on our previous work, providing further support that small LAP2 promoter is safe and effective suggesting that long-term transgene expression is possible in gene therapies targeting peripheral tissues.

## Supplementary Material

Supplementary Material

## Data Availability

The original contributions presented in the study are included in the article/Supplementary Material. Further inquiries can be directed to the corresponding author.
