# Supplementary material for: Persistent transgene expression in peripheral tissues one year post intravenous and intramuscular administration of AAV vectors containing the alphaherpesvirus latency-associated promoter 2
Source: Front Virol. Author manuscript; Available in PMC 2024 Apr 25. (PMC11044866; doi:10.3389/fviro.2024.1379991)
Supplement: Supplementary Material [file NIHMS1984882-supplement-Supplementary_Material.pdf]

Supplementary Material

Persistent transgene expression in peripheral tissues one year post intravenous and intramuscular administration of AAV vectors containing the alphaherpesvirus latency-associated promoter 2

Carola J. Maturana*, Esteban A. Engel

*** Correspondence:** Corresponding Author: maturana@princeton.edu

1.1 Supplementary Figures

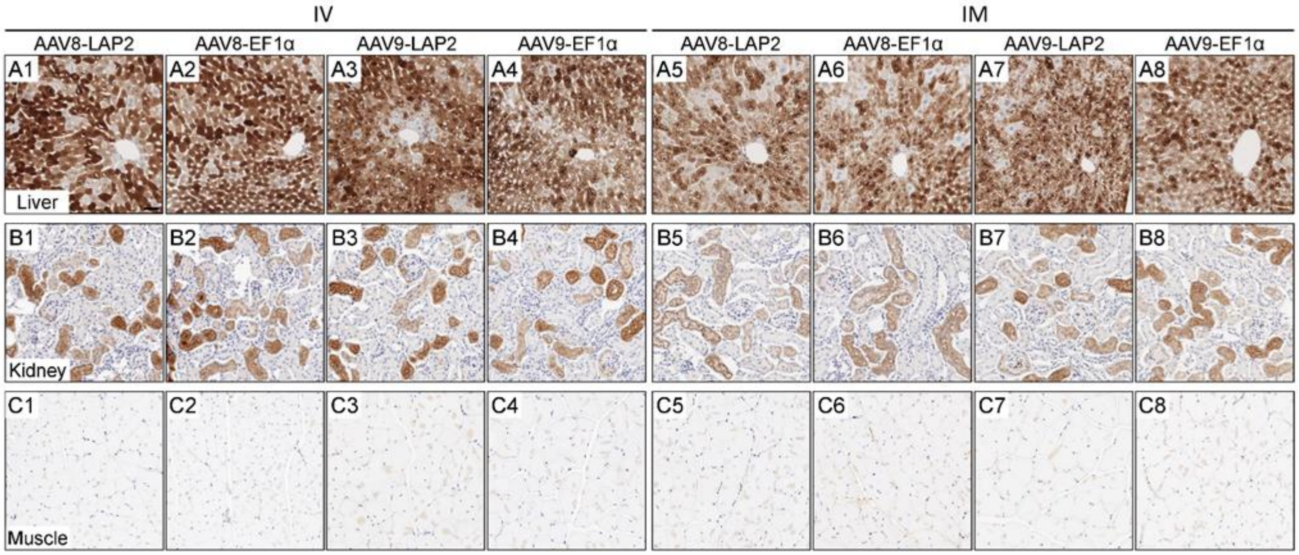

Supplementary Figure 1. Strong and widespread mCherry expression in the liver, kidney, and skeletal muscle of adult mice one-year post-administration of AAV8-/AAV9-LAP2 and AAV8/AAV9-EF1α promoters via local or systemic injection. Representative images document immunoreactivity to mCherry (brown) in (A) liver, (B) kidney, and (C) skeletal muscle tissue 400 days after the administration of AAV-LAP2 or AAV-EF1α. mCherry transgene expression in mice that received a single intravenous (IV) (A1–A4, B1–B4, C1–C4) or intramuscular (IM) (A5–A8, B5–B8, C5–C8) dose of AAV8-LAP2 or AAV9-LAP2 was compared with mice inoculated with AAVs carrying the EF1α promoter. Scale bar, 100 µm.

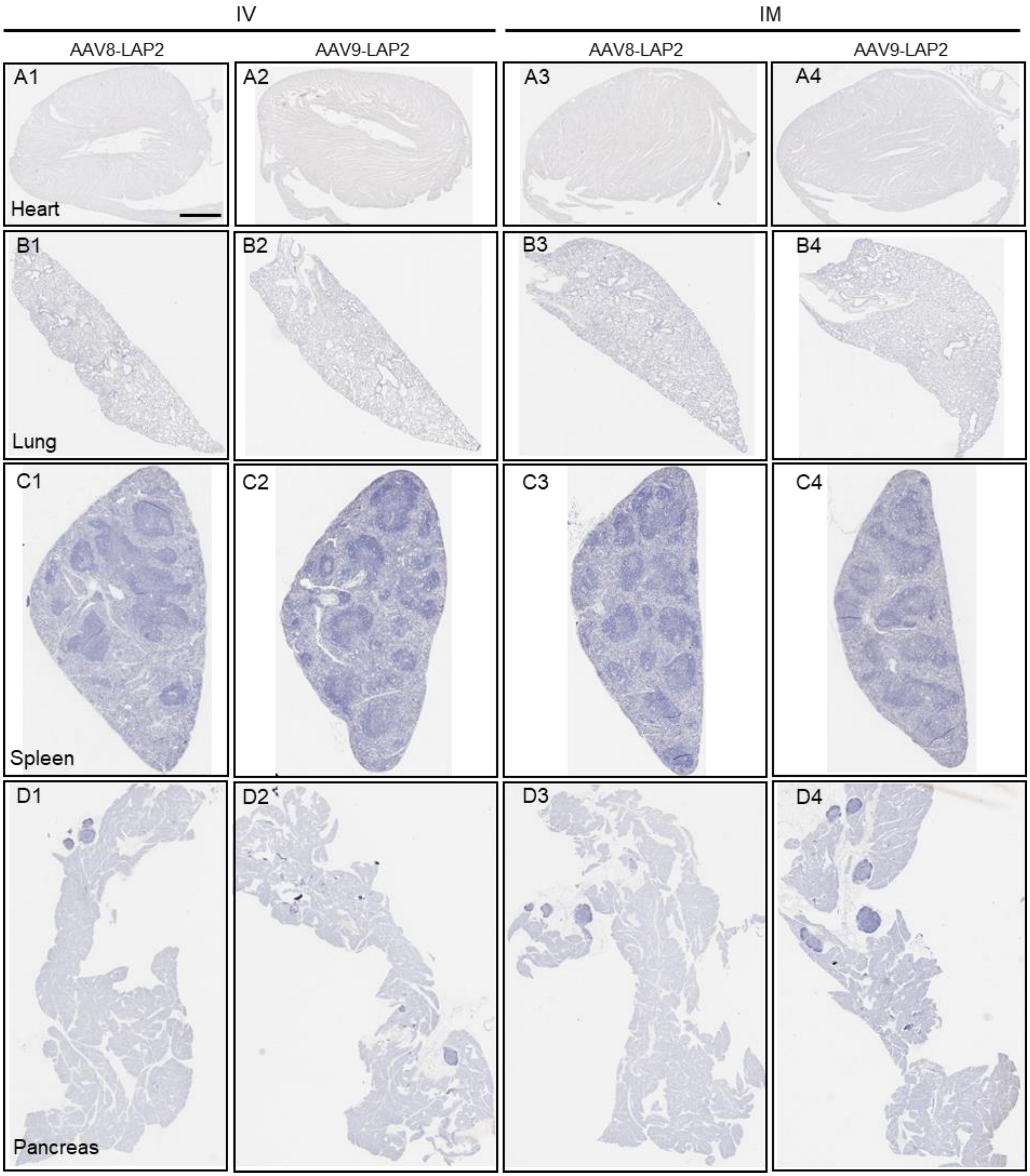

Supplementary Figure 2. Lack of mCherry expression in the heart, lung, spleen, and pancreas of adult mice after one year following local or systemic injection of 5 x 1011 gc dose of ssAAV8-LAP2 or ssAAV9-LAP2. Representative images demonstrate the absence of mCherry immunoreactivity (brown staining) in (A) heart, (B) lung, (C) spleen, and (D) pancreas tissue 400 days post-administration of AAV-LAP2. mCherry transgene expression in mice that received a single intravenous (IV) (A1–A2, B1–B2, C1–C2, D1–D2) or intramuscular (IM) (A3–A4, B3–B4, C3–C4, D3–D4) dose of AAV8-LAP2 or AAV9-LAP2. Scale bar, 2 mm.

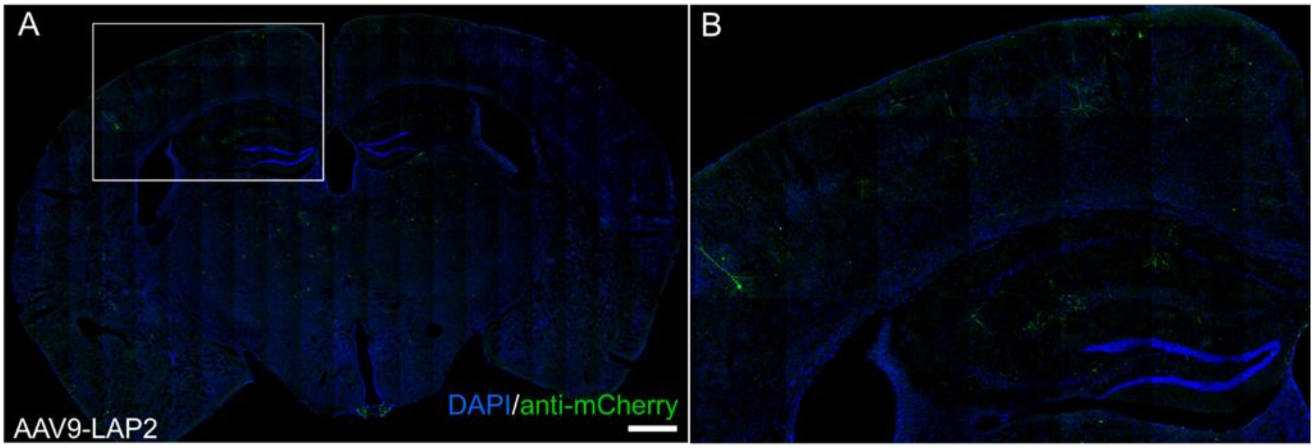

Supplementary Figure 3. Brain transduction efficiency of AAV9-LAP2 following systemic administration. Adult mice received an intravenous injection of 5 x 10^{11} gc dose of ssAAV9-LAP2-mCherry packaged into AAV9. Immunostaining for mCherry was performed 400 days post-injection. Representative images illustrate mCherry immunoreactivity (green) in (A) the coronal brain section and (B) a higher magnification image (white square) from (A). The image is a stacked confocal section. Scale bar: 1 mm.
